# Use of Computational Fluid Dynamics to Estimate Hemodynamic Effects of Respiration on Hypoplastic Left Heart Syndrome Surgery: Total Cavopulmonary Connection Treatments

**DOI:** 10.1155/2013/131597

**Published:** 2013-12-09

**Authors:** Jinlong Liu, Yi Qian, Qi Sun, Jinfen Liu, Mitsuo Umezu

**Affiliations:** ^1^Department of Cardiothoracic Surgery, Shanghai Children's Medical Centre, Shanghai Jiao Tong University School of Medicine, 1678 Dongfang Road, Shanghai 200127, China; ^2^Australian School of Advanced Medicine, 2 Technology Place, Macquarie University, North Ryde, Sydney, NSW 2109, Australia; ^3^ASMeW Lab, Centre for Advanced Biomedical Sciences, TWIns, Waseda University, 2-2 Wakamatsucho, Shinjuku, Tokyo 162-8480, Japan

## Abstract

Total cavopulmonary connection (TCPC), a typical kind of Fontan procedure, is commonly used in the treatment of a functional single ventricle. The palliative cardiothoracic procedure is performed by connecting the superior vena cava and the inferior vena cava to the pulmonary arteries. Due to the difficulty of direct study in vivo, in this paper, computational fluid dynamics (CFD) was introduced to estimate the outcomes of patient-specific TCPC configuration. We mainly focused on the influence of blood pulsation and respiration. Fast Fourier transforms method was employed to separate the measured flow conditions into the rate of breath and heart beat. Blood flow performance around the TCPC connection was investigated by analyzing the results of time-varying energy losses, blood flow distribution rate, local pressure, and wall shear stress distributions. It was found that the value of energy loss including the influence of respiration was 1.5 times higher than the value of energy loss disregarding respiratory influences. The results indicated that the hemodynamic outcomes of TCPC treatment are obviously influenced by respiration. The influence of respiration plays an important role in estimating the results of TCPC treatment and thus should be included as one of the important conditions of computational haemodynamic analysis.

## 1. Introduction

Fontan procedure was designed to treat complex congenital heart diseases (CHD) for patients with single ventricles, such as those suffering from tricuspid atresia and hypoplastic left heart syndrome (HLHS), which cannot be treated by biventricular repair [1]. During this kind of procedure, both the superior vena cava (SVC) and the inferior vena cava (IVC) are connected to the pulmonary arteries, thus allowing venous blood to flow directly from the body to the lungs via the pulmonary artery, bypassing the right ventricle. After Fontan and Baudet [2] introduced a nonanatomic correction of tricuspid atresia through innovative surgical approach in 1971, the original “Fontan” procedure has been modified into two main types of procedures—intracardiac (LT) lateral tunnel Fontan and extracardiac conduit (ECC) Fontan over the past 40 years.

Although survival rate after Fontan procedures has improved over the years, a number of unresolved questions continue to surround management and treatment [3]. Despite advancement in surgical techniques and medical therapies, pediatric cardiologists are still challenged by discussions regarding initial decision relative to treatment and long-term prognosis [4–6]. This is partly due to limited data available before and after the conduction of these therapies. Currently, many researchers and surgeons have been working to obtain such data for the improvement of the treatment of Fontan-type procedures based on the results of numerical investigations. These numerical techniques allow us to examine the effects of the geometry at the Fontan connection area and assess blood flow characteristics and energy loss (EL) associated with a given surgical design [7–11]. Numerous research papers have been published on the study of the Fontan procedure and its modifications, particularly, the total cavopulmonary connection (TCPC) procedure, such as Bove et al. [7], Migliavacca et al. [8], and Orlando et al. [9]. Meanwhile, many articles focused on the hemodynamic analysis of TCPC procedure. Sievers et al. [10] illustrated that the turbulence at the anastomosis of connection area was the reason for energy dissipation. Whitehead et al. [11] studied the power loss of TCPC connection area at exercise and the interaction between power loss and varying flow distribution to each lung under exercise conditions. However, most of these previous studies concentrated on the certain ratio of flow distribution within pulmonary arteries and the rate of EL during instantaneous rest or exercise. The physiological effects of blood flow pulsation and respiration were not considered. Previous clinical studies such as those of Rosenthal et al. [12], Pedersen et al. [13], and Hjortdal et al. [14] have indicated that pulmonary blood flow was obviously effected from breathing pressure. In recent years, some hemodynamic researchers began to investigate the effects of influence from blood flow pulsation and respiration, such as Marsden et al. [15, 16] and Itatani et al. [17], whereas little detailed information is available on numerical analysis to directly disclose the influence of respiration on hemodynamic characteristics in published works and papers. Marsden et al. [15] reported that respiration significantly affected Fontan flow rates and pressure. As a matter of fact, their article placed great emphasis on the analysis of respiration effects on the inflow at the SVC and IVC through the use of a quadratic polynomial form as respiration model. There was little investigation of the effect of respiration on pressure distribution at the inlets and outlets. In addition, although their studies have investigated the results of pressure decline through steady simulation with catheter-measured clinical data, the results were unable to provide useful boundary condition for CFD simulation to estimate the influence of respiration in independent patients. Itatani et al. [17] conducted the investigation of optimal conduit size of the ECC Fontan procedure by using basic geometry model. Although the influence of respiration was introduced into the simulation, constant pressure condition was applied at bilateral pulmonary arteries during expiratory phase, and pressure drop is assumed to be constant during inspiratory phase. The conditions have not precisely demonstrated the physiological effects from respiration. Marsden et al. [16] reported that they applied the coupled multidomain method with a lumped parameter three-element Windkessel model [18] at the outlets to investigate the influence of respiration. On the other hand, Stergiopulos et al. [19] and Segers et al. [20] have proved that the lumped parameter three-element Windkessel model overestimated the total arterial compliance and underestimated the characteristic impedance. The pulse pressure was predicted to be too high, and diastolic pressure wave was abnormal. Westerhof et al. [21] certified that the inflection point and the augmentation of pressure wave were not represented well by this model.

In the present research, we introduced the system of computational hemodynamic analysis, developed for first-stage HLHS surgery, Norwood procedure [22], to obtain the blood flow data at the TCPC connection area in detail, taking the influence of blood pulsation and respiration into account. The CFD methodology was applied to analyze the blood flow based on a patient-specific configuration acquired by magnetic resonance imaging (MRI), with physiologically realistic flow conditions measured in vivo through the utilization of ultrasound measurement in real time with electrocardiogram (ECG) at the SVC and IVC. Pressure conditions were obtained through the use of an intracardiac catheter with pressure sensors at the left pulmonary artery (LPA) and right pulmonary artery (RPA). We employed the method of fast Fourier transforms (FFT) to separate the measured blood flow through the different frequency of breaths and heart beats. We then compared the calculated results obtained imposing inflow boundary conditions for two cases: one considering both cardiac and respiratory pulsatility and the other considering only cardiac pulsatility. The results showed significant differences in EL, pressure, wall shear stress (WSS), and velocity magnitude. The aims of this study were to explore important issues of respiration effects on the hemodynamic analysis of TCPC procedure and to develop a method to improve the hemodynamic study of TCPC procedure by CFD technology.

## 2. Materials and Methods

### 2.1. Subject Selection and Clinical Information

In this study, the geometry of TCPC connection was extracted from a six-year-old female patient, who had been diagnosed with pulmonary atresia, ventricular septal defect, atrial septal defect, patent ductus arteriosus, and dexiocardia at birth. She underwent a Hemi-Fontan procedure at the age of two. During the intracardiac Fontan procedure, at four years of age, the IVC was connected to the RPA by an intracardiac lateral tunnel, and a 3 mm diameter fenestration was made on the lateral tunnel. With the consent of the parents and the approval of the local institutional review board and the regional research ethics committee, our patient-specific associated studies were approved.

**Figure 1:**
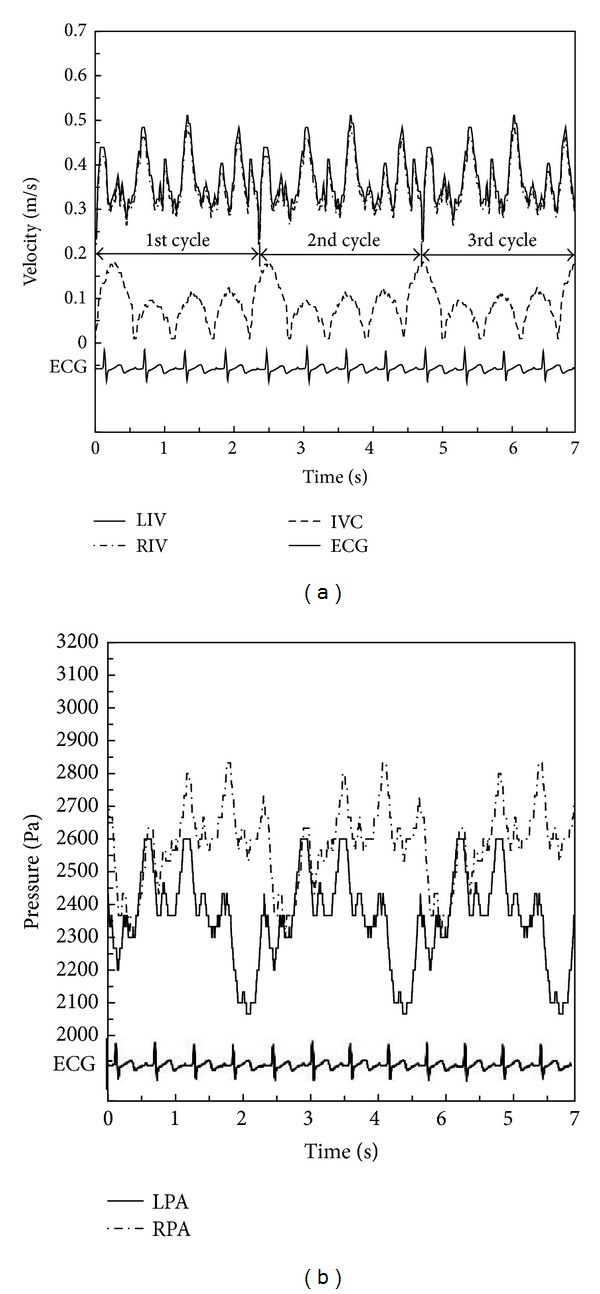
Clinical data as boundary conditions taking respiration into account. (a) Velocity data as inlet boundary conditions at the IVC, LIV, and RIV. (b) Pressure data as outlet boundary conditions at the LPA and RPA (IVC: inferior vena cava; LIV: left innominate vein; RIV: right innominate vein; LPA: left pulmonary artery; RPA: right pulmonary artery; ECG: electrocardiogram).

MRI and ultrasound were performed on the patient when she returned to the hospital for a follow-up medical examination. MRI and ultrasound results showed that the lateral tunnel fenestration made during the Fontan procedure had closed spontaneously. A series of continuous 4 mm thick MRI images were acquired on a 1.5 Tesla Signa Hispeed Scanner (GE Medical System, General Electric, USA) with a  256 × 192 pixel field of view to define the anatomies of Fontan connections. Moreover, the time-dependent velocity profiles of patient-specific physiological blood flow at the left innominate vein (LIV), right innominate vein (RIV), and IVC were obtained in real time through ultrasound measurement in with ECG. Pressure boundary conditions were measured in vivo real time through the use of an intracardiac catheter with pressure sensors. All data is depicted in Figure 1.

### 2.2. Model Reconstruction and Clinical Data Processing

**Figure 2:**
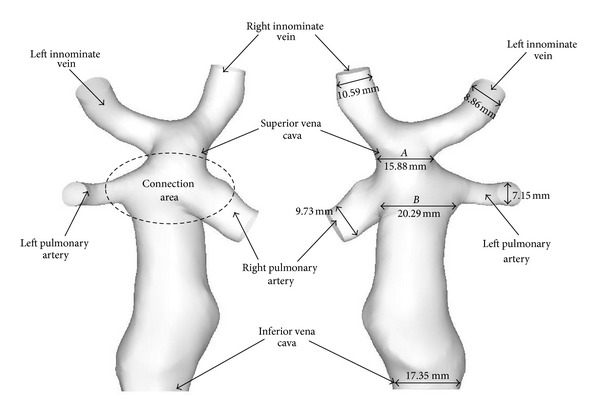
The patient-specific three-dimensional reconstructed configuration after surface smoothing.

Eighteen slices of cross-sectional fast imaging employing steady-state acquisition (FIESTA) sequence MRI images were used for patient-specific three-dimensional reconstruction of the vessels through commercial software Mimics 12.0. The accuracy of the reconstructed model had been checked against the geometry with an exacted measurement carried out by the original DICOM MRI slice files. A nonshrinking smoothing technique was employed to generate the numerical model for CFD simulation [23]. The geometry after smoothing is shown in Figure 2.

**Figure 3:**
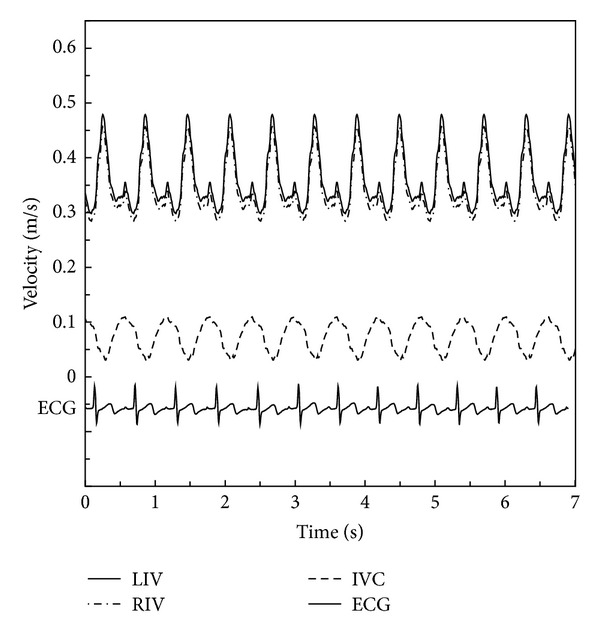
Velocity data as inlet boundary conditions only including cardiac pulsatility (IVC: inferior vena cava; LIV: left innominate vein; RIV: right innominate vein; ECG: electrocardiogram).

The velocity result files at LIV, RIV, and IVC, acquired through ultrasound measurement, were stored in ASCII format as boundary conditions for the CFD simulation. The velocity results contained the respiratory cycle data. We used the FFT methodology to separate the interaction of respiration by the different frequency between breath and heart beat through the use of commercial software MATLAB 7. Consequently, the present study could be conducted under the following conditions: (I) pulsatile flow without considering the influence of respiration and (II) pulsatile flow with the influence of respiration. Detailed information of blood velocity after FFT separation is displayed in Figure 3. One cycle of breath includes four heart pulsatile circulations.

### 2.3. Governing Equations

The flow simulation was based on the Navier-Stokes (N-S) momentum equation and continuity equation defined as follows:

(1) ∂∂t(ρui)+∂∂xj(ρuiuj) =−∂p∂xi+∂∂xj[μ(∂ui∂xj+∂uj∂xi)]+fi,∂ρ∂t+∂∂xj(ρuj)=0,

where *i*, *j* = 1, 2, 3, *x*_1_, *x*_2_, *x*_3_ represent coordinate axes, *u*_*i*_, *u*_*j*_, and *p* are the velocity vectors and the pressure at any point in the flow domain, *ρ* and *μ* are blood density and viscosity, and *t* is time. The term *f*_*i*_ expresses the action of body forces.

Due to the relatively large sizes of the vessels compared to individual blood cells [24] and the great shear stress typical within larger arteries [25], the blood was assumed to be a Newtonian fluid with a constant density (*ρ* = 1060 kg/m^3^) and viscosity (*μ* = 4.0 mPas) [26–28], while the body forces were omitted. Therefore, the N-S equations are turned into the following form:

(2) $$\begin{array}{c} \rho \frac{\partial U}{\partial t}+\rho \left(U\cdot \nabla \right)U-\mu \nabla^{2}U+\nabla p=0,\\ \nabla \cdot U=0, \end{array}$$

where  *U* = *U*(*u*_1_, *u*_2_, *u*)  is the velocity vector.

The Reynolds number (Re), the ratio of the inertial forces to the viscous forces in the flow, is defined by

(3) Re=ρUDμ,

where  *D*  is the characteristic length.

In this TCPC case, time-averaged velocity was 0.3419 m/s with maximum Re number of 802.53 at Condition I. At Condition II, considering the influence of respiration, the maximum Re was lower than 1000, which was calculated at the peak of the velocity wave at Condition II. Therefore, the flow is able to be described as a laminar flow at both conditions of this study. From the clinical viewpoint, the intracardiac lateral tunnel reduced atrial enlargement due to the decreasing of right atrial surface space. De Leval et al. [29] and Mavroudis and Backer [30] investigated that the flow passing through the baffle was laminar flow, which improved the hemodynamic performance of the Fontan circuit flow and reduced the EL within the dilated right atrium.

### 2.4. Mesh Generation

**Figure 4:**
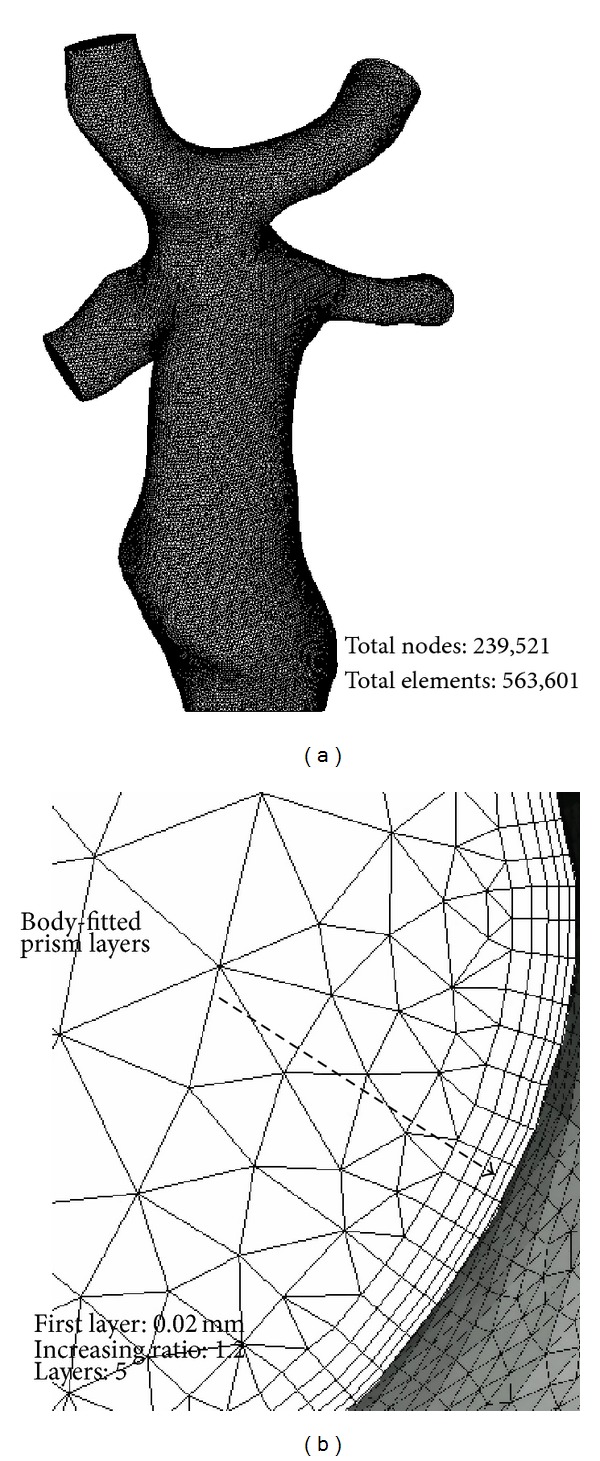
Mesh information. (a) Calculation domain with grids. (b) Body-fitted prism layers.

The grid-generation software ANSYS-ICEM 12.1 was utilized to discretize the computational domain. In order to accurately measure WSS at near-wall regions, the body-fitted prism layers were generated near the vessel walls to improve the resolution of the relevant scales in fluid motion. There were five layers generated with an average nodal space, increasing by a ratio of 1.2. The distance from the first layer to the vessel surface was fixed at 0.02 mm. From the centre of the vessels to the prism inner layer, tetrahedral grids in varying sizes were utilized. To verify the reliability, grid independence of the WSS [31] was checked by calculation with finer meshes, where the maximum cell size was decreased to 0.2 mm, with no consequent change occurring in the results of WSS. The generated meshes are shown in Figure 4.

**Figure 5:**
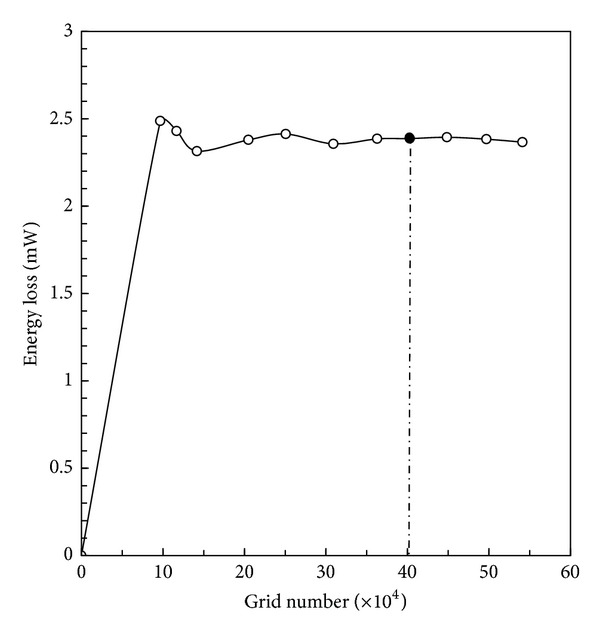
Results of grid independent verification.

Considering that the quality of CFD results was highly dependent on grid resolution and boundary conditions, a series of grid-dependent validations were performed by extending the domain length at inlets and outlets. As shown in Figure 5, when the grid number at the research domain was around 400,000, the EL started to converge into a constant. Therefore, accurately reliable results could be obtained with a total of 563,601 finite elements and 239,521 nodes, as utilized in the current study.

### 2.5. Boundary Conditions and Calculation

According to the research of Migliavacca et al. [32], the different velocity profiles adopted at the inlets only have minor effects on blood distribution into the lungs in the TCPC simulation. Though there were slight differences, the volume flow curves calculated under zero pressure at the outlets practically coincided with the curves from in vivo measurements. Thus, the pulsatile velocities shown in Figure 3 were applied as the inlet boundary conditions (BCs), and a zero pressure condition was used at the outlets in the simulation of Condition I. Different boundary conditions were utilized in the simulation of Condition II. The time-dependent velocities and pressure displayed in Figure 1 were used as the inlet BC and outlet BC. For these simulations, we assumed the rigidity of vessel wall surfaces.

The finite volume solver package ANSYS-FLUENT 6.3 was employed to solve the fluid equations. The N-S equations were solved by the transient solution method, and the Adams-Bashford method was applied for the second-order transient solution of the time-dependent N-S equations. The terms of pressure and momentum in the equations were discretized using a second-order upwinding scheme, and the convergence criteria were to reduce the residuals of continuity and momentum equations to 10-5. A subroutine based on the C programming language was written in order to create a user-defined function for the Fluent code to impose time-dependent velocities as the inlet BCs and pressure as the outlet BCs. The vessels were assumed as rigid and no-slip surfaces at both Conditions. In order to obtain a periodic solution, six heart cycles for Condition I and three times of breath (12 heart beats) for Condition II were carried out in total, respectively. To obtain accuracy, calculation time step was set to 0.0001 seconds according to the calculation of the Courant number, defined as follows:

(4) $$\begin{array}{c} C_{r}=\bar{u}\frac{\Delta t}{\Delta I}, \end{array}$$

where$\;\bar{u}\;$is average velocity,  Δ*t*  is maximum time step size, and  Δ*I*  is dimension of grid cell. In total, 36,000 and 68,000 steps were calculated for Condition I and Condition II, with the calculation time set to nine days and eighteen days, respectively. All of the computations were performed on a workstation with a Windows XP operation system. The workstation was equipped by double CPUs: Intel (R) Pentium III Xeon (R) 3.0 GHz processors, with 16.0 GB RAM memory, and a 64-bit Windows XP operation system.

### 2.6. Flow Distribution Ratio and Energy Loss

To evaluate the effects of respiration on hemodynamics, quantitative indexes were defined, including the flow distribution ratio (FR) and control volume energy loss (EL). The FR used to evaluate the outcomes of TCPC procedure on the balance of blood distribution between the LPA and RPA is given by

(5) $$\begin{array}{c} \text{FR}=\frac{Q_{\text{LPA}}}{Q_{\text{inlet}}}\times 100\%, \end{array}$$

where  *Q*_LPA_  is the flow in the left pulmonary artery and  *Q*_inlet_  is the sum of the IVC, LIV, and RIV inlet flows.

The control volume (CV) EL, also named control volume power loss, is defined by

(6) EL=∑t[∑i(Pi+12ρUi2+ρ∂Φi∂t)Qi−∑o(Po+12ρUo2+ρ∂Φo∂t)Qo],

where  *i*  is element number at inlet and *o* is element number at outlet. Inlet boundaries include the IVC, LIV, and RIV, while the outlet boundaries are the LPA and RPA. ∂Φ/∂*t* denotes the partial derivative of the velocity potential Φ with respect to time *t*, *U* = |∇Φ| is blood velocity, and *P* and *Q* define static pressure and volume flow rate, respectively.

## 3. Results

**Figure 6:**
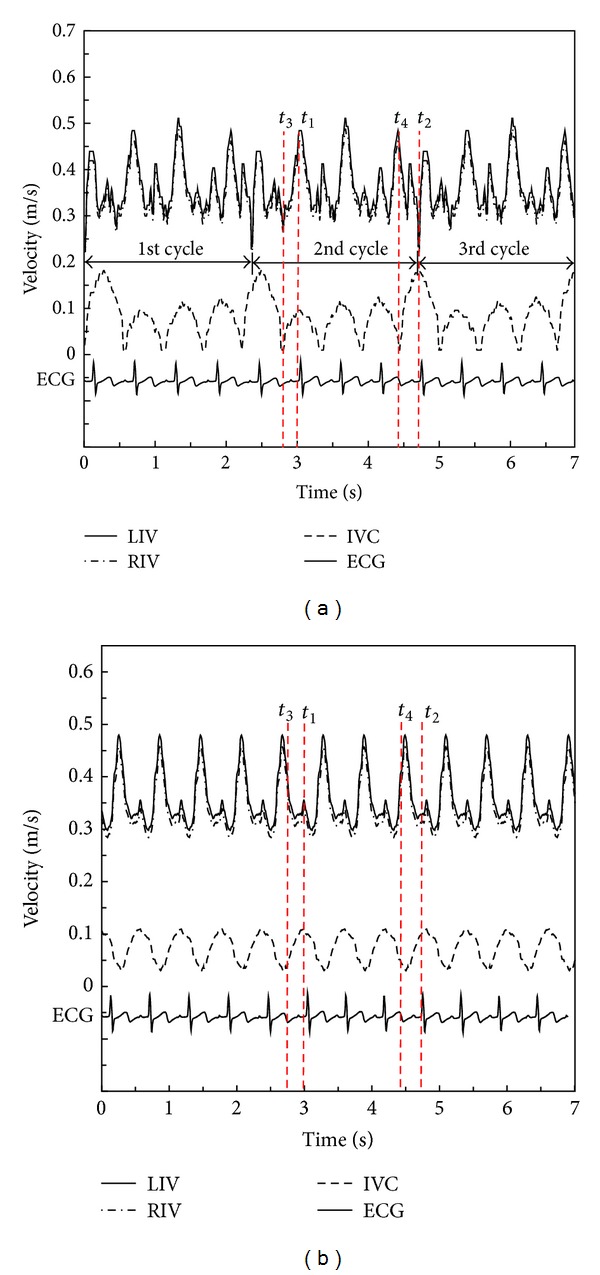
Four selected time steps for hemodynamic comparison (*t*_1_, *t*_2_, *t*_3_, and *t*_4_). (a) Inlet velocity conditions taking respiration into account. (b) Inlet velocity conditions only including cardiac pulsatility (IVC: inferior vena cava; LIV: left innominate vein; RIV: right innominate vein; LPA: left pulmonary artery; RPA: right pulmonary artery; ECG: electrocardiogram).

We selected four different time steps (*t*_1_, *t*_2_, *t*_3_, and  *t*_4_) to compare the hemodynamic characteristics between the two conditions. The detailed information of the four time steps is given in Table 1. The boundary conditions at these steps are shown in Figure 6.

**Figure 7:**
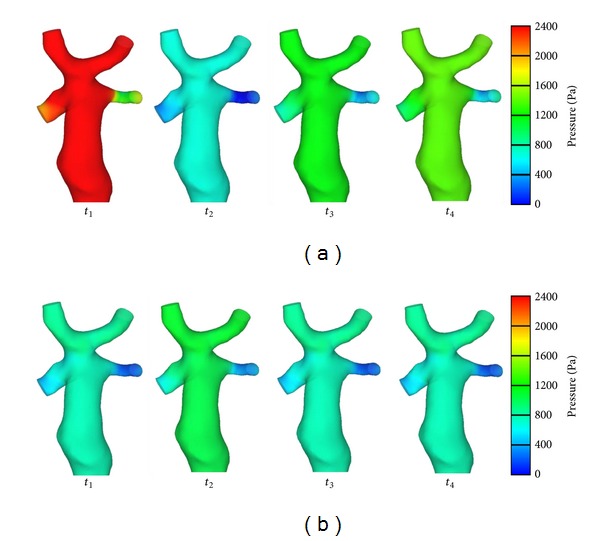
Pressure distribution (Condition II (a) and Condition I (b)).

In order to compare the difference of pressure distribution between two boundary conditions, the relative pressure, which was calculated by using boundary Condition II, was reduced to the same level of boundary Condition I (minimum diastolic pressure at  PLPA = 0). The modified pressure results were shown in Figure 7. Due to our interest in relative pressure, the pressure modification did not influence the results of EL calculation. The pressure was much higher at Condition II than that at Condition I at *t*_1_, *t*_3_, and *t*_4_ while being lower at *t*_2_. The influence of respiration was believed to be significant for pressure distribution. We also found relatively lower pressure zones at the entrances of LPA and RPA, with the size of these zones altered with each subsequent time step.

**Figure 8:**
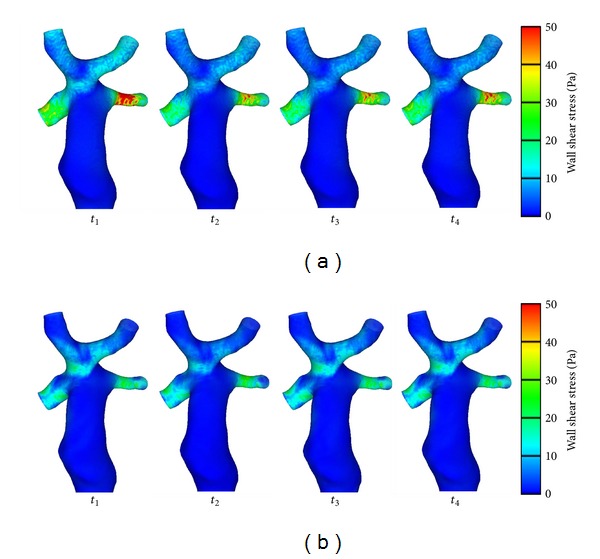
Results of wall shear stress (Condition II (a) and Condition I (b)).

WSS is displayed in Figure 8. We observed relatively higher WSS distribution in three zones on the configuration at both conditions: the SVC connection area, the RIV, and the RPA. Examining the geometry, the distorted spatial configuration of vessels created in the surgery might be one of the main reasons. The highest value of WSS was found at the LPA at Condition II. It indicated that greater energy was lost at this location. Also, the size and location of these high-WSS zones changed instantaneously with the four time steps. Because a close relationship exists between WSS and velocity gradient (*τ*_wall_ = −*μ*∂*U*_*x*_/∂*y*|_*y*=0_, where *τ*_wall_ represents WSS, *μ* is blood viscosity, ∂*U*_*x*_/∂*y* is velocity gradient, *U*_*x*_ is velocity of the fluid near the boundary, and *y* is the height above the boundary), the velocity gradient was believed to have diversified at these zones with each subsequent time step. On the other hand, we found that a relatively low WSS existed at the SVC at Condition II and that the lower value most likely weakened the influence of blood flow on endothelial layer function at this zone. Thus, the influence of respiration should be counted as one of the important factors in the calculation of the results, and this influence may create minor oscillations of WSS at Condition II.

**Figure 9:**
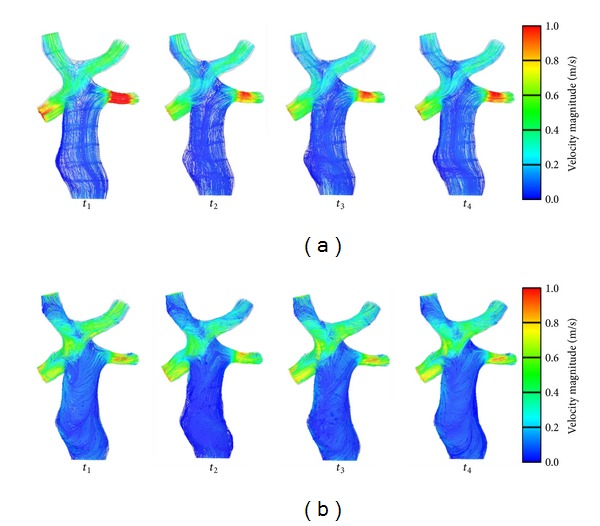
Results of streamlines (Condition II (a) and Condition I (b)).

Figure 9 depicts the streamlines at each time step. High-speed zones distributed at the LPA and RPA at both conditions. The highest value of velocity was found at the LPA at Condition II, meaning that respiration affected the pulmonary blood flow through this increase of velocity. At the confluent location, two flows from LIV and RIV were blended, and a low-speed area was observed at both Condition I and Condition II. Moreover, a relatively lower velocity was found in the confluent area at Condition II. This implied that greater EL was generated when the two flows encountered and passed through the SVC connection area. An unstable blended flow occurred at the connection area between the IVC and SVC.

**Figure 10:**
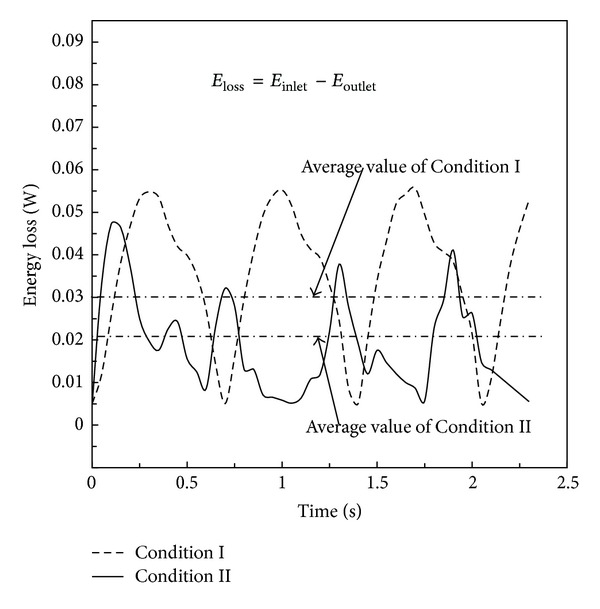
Results of energy loss at Condition I and Condition II.

Time-dependent EL and time-averaged EL were calculated as shown in Figure 10. The EL was lower at Condition II. The maximum value of time-dependent EL was approximately 0.04725 W at Condition II, lower than that at Condition I, 0.05581 W. Furthermore, the time-averaged value of EL at Condition II was approximately 1.5 times lower than that at Condition I. This implied that the blood flow could obtain energy from respiration to complement losses caused by flow, confirming the conclusion of the previous study that spontaneous breathing provided additional energy for blood flow [33, 34]. More specifically, the maximum value of this difference was around 0.05 W. Unlike the regular curve shape at Condition I, the curve of EL fluctuated at Condition II during the whole breath cycle. A big wave followed some small waves. It indicated that inspiration and expiration had different influence on EL. When the patient inspired, the maximum value of EL appeared. From the time-dependent EL, we found some evidence of the influence of respiration on EL. Detailed analysis will be part of future work on the investigation of the time-dependent EL during both inspiration and expiration. This will likely become one of the methods for the patient's clinical examination, through the calculation of real-time EL in blood flow, with the influence of respiration.

**Figure 11:**
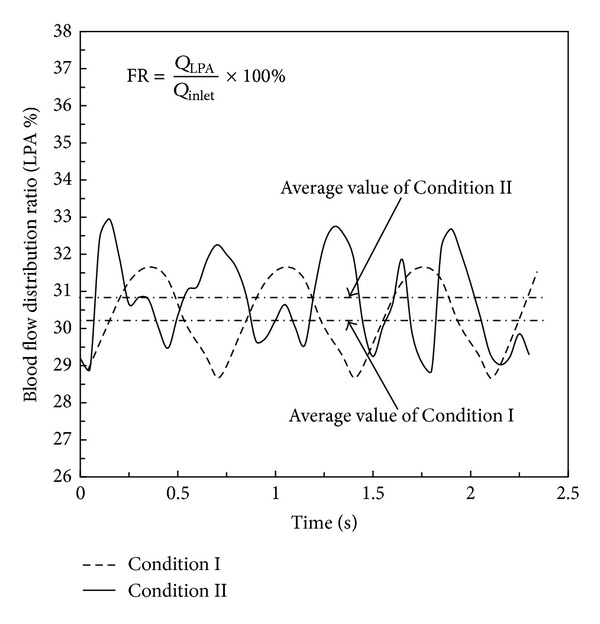
Results of flow distribution ratio at Condition I and Condition II at LPA (FR: flow distribution ratio; LPA: left pulmonary artery).

Figure 11 describes the percentages of flow ratio distributed at the LPA. The temporal value of the proportion at the LPA was different at each subsequent time step, at both conditions. The instantaneous value of the percentage varied between 29% and 33% at Condition II in one heart cycle, while the value at Condition I ranged from 28.9% to 31.5%. However, the time-averaged value of FR at both conditions was almost the same, around 30%. There was approximately only a 0.6% difference between both values. The results revealed that respiration was not the main influence on the results of the mass flow distribution and that the time-averaged ratio of mass flow distribution between the LPA and RPA was close to 3 : 7 in this patient-specific case.

## 4. Discussion

Because the TCPC connection area was located in the thoracic cavity and was connected to the lungs through pulmonary arteries, the effect of respiration may have had a great influence on the hemodynamic features in the anastomotic region. In the present study, the computational hemodynamic analysis methodology was used to analyze the characteristics of local blood flow by evaluating the influence of respiration in pulsatile simulation. The accuracy of computation methodology on pulsatile simulation has been discussed in our previous work [22].

Patient-specific pressure data was used as boundary conditions to estimate the hemodynamic influence of respiration. Although we noted that lumped parameter methods have been reported in some cardiovascular hemodynamic literatures, they were still short in evidence for the confirmation of its accuracy in vivo. The method cannot model higher spatial-resolution aspects without introducing sufficient elements [35]. Further validation is still required to confirm its suitability to reflect actual patient-specific hemodynamic situations, particularly in regards to congenital heart disease patients. A series of patient follow-up studies are being continuously performed in our hospitals. We believe that these in vivo data may be available to validate boundary conditions, including the lumped model, in the future.

Local pressure, WSS, streamlines, EL, and FR at the region of TCPC anastomosis were analyzed by calculations at Condition I and Condition II. The results showed that the regional flow patterns within the connection area, which was related to the distribution of local pressure, WSS, and streamlines, differed from those calculated when the influence of respiration was not taken into account, particularly for the size of low-speed areas and local vortical flow. The spatial conformation of the LIV, created in surgery, led the blood to flow directly into the anterior part of the SVC and then into the RPA. Blood flow from the RIV directed to the posterior part of SVC and split into the LPA and the RPA. The flow from the IVC interacted with that from the LIV and RIV and then directed into the LPA and RPA. A low-velocity flow region existed at the LIV-RIV bifurcation, and EL occurred as a result in the area. Moreover, due to the different frequencies between breath and heart beat, the unstable blood flow was shown to be delayed slightly at the IVC at Condition II. This could be explained by the delaying of the peak value at the inlet when respiration was taken into account. Respiration changed the pulsatile periodicity of blood flow. Meanwhile, the intensity of vortical flow at the IVC domain was slightly decreased. The salient part generated in the surgery near the entrance of IVC should be the reason as to why a low-speed area and flow recirculation existed in this region.

We believe that WSS must be discussed at instantaneous conditions. The specific times, selected in this study to compare instantaneous WSS, followed usual cardiovascular applications: systolic peak and diastolic bottom (see Table 1). Furthermore, it is well known that WSS is a vector value. Thus, time-averaged WSS may result in a loss of significance due to alternations of the WSS between positive and negative values.

At conditions of high WSS, the WSS may influence the development of vessels following the TCPC Fontan procedure. The properties of the vessels must be taken into consideration when analysing local hemodynamics. On one hand, WSS also affects normal cardiovascular growth through gene expression [36] and activates blood-forming stem cells [37]. The long-term effects of WSS, on the other hand, include vascular remodeling and endothelial damage. By detailed examination of WSS between the two conditions at instantaneous time points, we found that respiration can weaken the effect of WSS on vessel walls. These results indicated that WSS may be overestimated if the calculation fails to consider the influence of respiration in hemodynamic analysis.

In Figure 10, the values of both time-dependent EL and time-averaged EL at Condition II were lower than those at Condition I. One of the possible reasons was that respiration could deliver energy through the transmission of pressure waves. Furthermore, with the influence of respiration, the unstable flow in the confluence region was attenuated, and the direct head-to-head collision of the blood flow from the LIV, RIV, and IVC was decreased. Therefore, relatively low EL was displayed at Condition II, as shown in Figure 10.

The EL was used as an effective indicator in many papers to evaluate the efficiency of the operative designs. In this study, we applied EL to reveal the efficiency of the TCPC connection. When unfavorable vessel connections result in flow separation and collision, EL may exhibit sharp increases. Furthermore, we should emphasize that the EL was calculated at a single pulse. If it was assembled accordingly to days or years, the EL will be of nonnegligible number for the newborn patient. It can be utilized as an indicator to evaluate the outcomes of surgically created Fontan circulation. From a hemodynamic point of view, energy loss fully describes the hemodynamic severity, as well as any impacts on the pumping ventricle. High energy loss thus impacts the pumping ventricle which, together with the peripheral vasculature, adapts itself to work harder to overcome the added drag so as to meet the functions of the circulatory system. Thus, this almost certainly results in chronic heart failure as a secondary disease [38]. Therefore, EL is a vital factor in the estimation and evaluation of hemodynamic characteristics.

Time-dependent effects of respiration on the FR are shown in Figure 11. Compared with the average value of FR at the two conditions at the LPA, no noticeable difference occurred when the influence of respiration was taken into account. It could be concluded that respiration had little effect on the FR in the patient-specific TCPC study. Whether the influence is universal or not, more patient-specific data is required for examination in future studies. Currently, estimation of FR enables to evaluate the balance of blood flow distribution after Fontan procedure so as to enhance clinical diagnosis for the long-term patient's following therapies.

The important issue of this study was essential for future correlative studies in the design of Fontan-type procedures. The influence of respiration should be considered, especially in the analysis of local hemodynamic characteristics. To estimate quantitative analysis more accurately and make regional flow features approach the realistic conditions, we advocated that the influence of respiration should better be included in numerical simulations.

There are most likely two limitations of the present study which should be considered. Firstly, this study used the rigid vessel model in the calculation of both conditions. Therefore, the interaction of respiration and vessel compliance was not taken into account. Although recent studies illustrated that the method of fluid-structure interaction (FSI) scheme was available [39], the complexity of clinical measurements of vessel properties and the lack of well-established validation methods for FSI simulation are still problems required to be solved in future work. Secondly, the present study was a single patient-specific research. More cases should be investigated in the following studies.

## 5. Conclusion

Based on the present study, the conclusion could be drawn that the local hemodynamic characteristics at the TCPC connection area were greatly influenced by respiration, including the distribution of static pressure, WSS, and streamlines. Respiration is one of the important physiological factors contributing to the weakening of the effect of WSS on vessel walls and the offset of EL caused by blood flow. However, there was no obvious difference in the time-averaged distribution ratio of blood flow to pulmonary arteries when the influence of respiration was considered. Furthermore, the effects of respiration should better be considered in the correlative studies for the purpose of physiological patient-specific TCPC investigation. Numerical analyses based on in-depth examinations of the physiological flow conditions of patients are required in order to evaluate the outcomes of the Fontan therapy. Considering the results of the current study, greater emphasis should be placed on the influence of respiration during the numerical simulation of TCPC Fontan-type procedures.

In future studies, alongside continual study of the issues mentioned above, our work will also concentrate on the analysis of the influence of respiration and vessel compliance. The method of obtaining this clinical data will be the crucial point in the investigation.

## Figures and Tables

**Table 1:** The definition of four selected time steps at Figure 6(a).

Velocity information		
Time step	IVC	LIV and RIV
*t*_1_	Peak	Peak
*t*_2_	Peak	Bottom
*t*_3_	Bottom	Bottom
*t*_4_	Bottom	Peak

## APPENDIX

### Notation

μ: Dynamic viscosity
ρ: Density
τwall: Wall shear stress
ΔI: Dimension of grid cell
Δt: Maximum of time step size
fi: Body forces
i, j: Coordinate axes
t: Time
u¯: Time-averaged velocity
ui, uj: Velocity vectors
Cr: Courant number
D: Characteristic length
EL: Energy loss
FR: Flow distribution ratio
P: Pressure
Q: Volume flow rate
Re: Reynolds number
U: Velocity vector
WSS: Wall shear stress.
